# The Efficacy and Safety of High-Frequency Deep Sclerotomy in Treatment of Chronic Open-Angle Glaucoma Patients

**DOI:** 10.1155/2019/1850141

**Published:** 2019-11-16

**Authors:** Mokhtar Mohamed Ibrahim Abushanab, Ayman El-Shiaty, Tarek El-Beltagi, Shaymaa Hassan Salah

**Affiliations:** ^1^Research Institute of Ophthalmology, Cairo, Egypt; ^2^Kasr Alainy, Faculty of Medicine, Cairo University, Cairo, Egypt

## Abstract

**Purpose:**

Highlights the high-frequency deep sclerotomy as a competing ab interno surgery in open-angle glaucoma patients and evaluates the efficacy of high-frequency deep sclerotomy in lowering IOP of chronic open-angle glaucoma patients, in addition to the evaluation of the safety of the procedure on the corneal endothelium.

**Design:**

A prospective study. It was performed in the Research Institute of Ophthalmology, Cairo.

**Participants:**

43 eyes were enrolled for the study. All eyes were diagnosed with chronic open-angle glaucoma.

**Methods:**

The procedure was performed using the abee® glaucoma tip of Oertli phacoemulsification machine (OS3, Switzerland) and goniolens performing nasal six pockets penetrating 1 mm deep into the trabecular meshwork and Schlemm's canal. Patients were followed up regarding vision and intraocular pressure (IOP) for 9 months. Endothelial cell count and coefficient of variation were calculated using specular microscopy preoperatively and 9 months postoperatively.

**Results:**

The mean preoperative IOP was 31.4 ± 4.71 mmHg (range: 22 to 42 mmHg). All patients were followed up for nine months. The mean IOP was 19.06 ± 1.907 mmHg (range: 15 to 26 mmHg) by the end of the study. The reduction across the follow-ups was statistically significant (*P* < 0.0001). The percentage of total success was 90.7% (39 eyes). Four eyes (9.3%) had IOP > 21 (failed) after surgery and continued on antiglaucoma therapy.

**Conclusion:**

High-frequency deep sclerotomy is a safe and promising minimally invasive procedure which successfully achieves a lower IOP for chronic open-angle glaucoma patients, but not adequate for advanced glaucoma patients. This trial is registered with PACTR201910823824561.

## 1. Introduction

Subscleral trabeculectomy (SST) is considered the standard technique when surgery is needed to lower intraocular pressure (IOP) in chronic open-angle glaucoma patients [1]. Nevertheless, the development of nonpenetrating glaucoma surgeries, such as deep sclerectomy [2], viscocanalostomy [3], and canaloplasty [4], have evolved to bypass the resistance in the pathologic inner wall of Schlemm's canal without entering the anterior chamber and leaving the intact trabeculo Descemet membrane [5].

Newer surgical techniques were introduced to overcome the transconjunctival approach and to increase the safety of glaucoma surgeries, which were collectively named minimally invasive glaucoma surgery (MIGS). Some of these procedures work via enhancing the filtration through Schlemm's canal like trabectome [6], iStent [7], Hydrus implant [8], and high-frequency deep sclerotomy (HFDS) [9]. HFDS utilizes the reusable high-frequency diathermic probe tip called abee tip (abee® glaucoma tip, Oertli Instrument, Switzerland) for this procedure [9]. It produces a communicating canal from the trabecular meshwork (TM) and Schlemm's canal (SC) to the sclera using the characteristics of high-frequency power to dissipate the tissues around the tip as Dr. Pajic explained [10]. This type of surgery needs to be recalled for investigation for its safety and IOP lowering effect.

The aim of our study is not only to evaluate the short-term efficacy of the procedure in lowering the IOP but also to evaluate the risk of HFDS on back of the cornea (endothelial cell density and coefficient variation) using specular microscopy.

## 2. Patients and Methods

The present study followed the tenets of the Declaration of Helsinki. It was approved by the ethics committee of the Research Institute of Ophthalmology (R.I.O), and informed consents were obtained. The study is an interventional case-series study conducted at R.I.O. The patients were randomly recruited from the ophthalmology outpatient clinics in R.I.O in the period from July 2016 till December 2018. All eyes included in the study were suffering from chronic open-angle glaucoma and were indicated for surgery due to either progression of field defect and raised intraocular pressure above 21 mmHg in spite of maximum medical treatment or for patients who were not compliant patient to their medical treatment. Patients with congenital, traumatic, neovascular, and uveitic glaucoma, patients with prior penetrating and nonpenetrating glaucoma surgeries or laser therapy, and eyes with corneal opacity precluding visualization of the angle were excluded from the study. The baseline ophthalmic examination involved best corrected visual acuity (VA) testing using the Snellen decimal scale. IOP was measured using applanation tonometer. Slit-lamp examination was performed to evaluate the corneal condition and anterior chamber depth, and gonioscopy was undergone using four mirror gonioscopic lens. Lens was examined to determine whether the patient is phakic or pseudophakic and grade of cataract if present (according to Lens Opacification Classification System III (LOCS III)). Fundus examination was performed for optic disc evaluation. Endothelial cell count was evaluated using specular microscopy (NIDEK CEM-530, Japan). The number of glaucoma medications used was also documented.

Postoperative ophthalmological follow-up was carried out the *first day*, at the end of the *first week* and *second week* postoperatively, and then regular follow-ups were scheduled as follows: *first month, three months, six months, and nine months*. abee® *Glaucoma* Tip of Oertli Phacoemulsification Machine.

The tip of the probe is bent 15° posteriorly and is 1 mm in length, 0.3 mm in height, and 0.6 mm in width. Its outer diameter is 0.9 mm. The tip's dimensions make it one of the MIGS [10].

### 2.1. Surgical Technique

**Figure 1:**
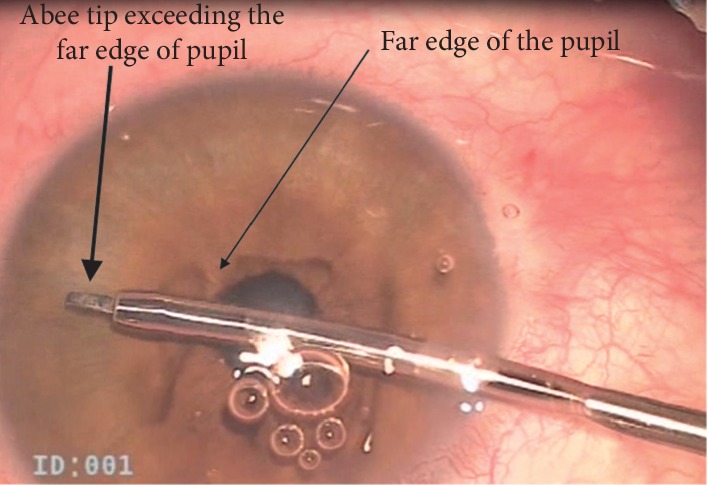
abee® tip exceeds far border of the pupil before applying goniolens.

**Figure 2:**
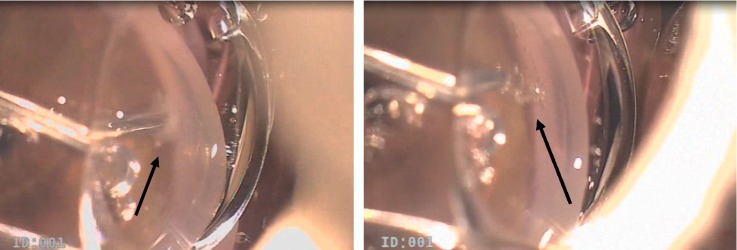
Bubbles formed during surgery. Black arrows demonstrate the bubbles.

Following local peribulbar anesthesia, two clear corneal incisions were constructed using 19G MVR blade 120 degrees apart located in the upper temporal and upper nasal quadrant. Evacuation of aqueous in some cases lowers IOP, so blood passes to Schlemm's canal making visualization in difficult cases easier. High-density cohesive ophthalmic viscoelastic device (OVD) was injected to fill the anterior chamber. Then, the viscoelastic material was applied to the surface of the cornea. The abee® glaucoma tip of Oertli phacoemulsification machine (OS3, Switzerland) was introduced through one of the side ports and advanced towards the opposite iridocorneal angle (Figure 1). A four mirror goniolens was used to visualize the angle, introducing the abee® glaucoma tip towards TM. The goniolens was applied after the tip of the probe passes the far edge of the pupil. The abee® glaucoma tip was pushed about 1 mm towards the sclera through the TM and Schlemm's canal by pressing the Oertli machine foot pedal till hearing the alarming beep three times. Multiple bubbles at the tip were noticed, and then, the abee® tip was pulled away (Figure 2). A total of six pockets in the nasal sclera were created. Each pocket was 0.3 mm thick and 0.6 mm wide. Viscoelastic was washed, and side ports were hydrated.

Postoperative medical treatment was tobramycin/dexamethasone drops, either as a fixed-dose combination or separately four times a day for four weeks and pilocarpine 2% eye drops for four weeks.

The postoperative evaluation of the cases was done at a regular schedule: first day, first week, second week, first month, and third, sixth, and ninth months. The postoperative IOP and VA were measured in every follow-up visit. We considered total success if IOP<21 mmHg without treatment and qualified success if IOP<21 mmHg with ocular hypotensive medical treatment, and failure was defined as IOP>21 mmHg even with use of full ocular hypotensive medications, with the necessity for further glaucoma surgery.

### 2.2. Statistical Analysis

Statistical analysis was done by IBM SPSS v21.0 statistical software (IBM Corporation, New York, USA). Descriptive data were calculated and was introduced as mean ± standard deviation (±SD), median and range, or frequencies and percentages when appropriate. For assessing the association between categorical data, the chi square (*χ*^2^) test was performed. Comparison of numerical variables between preoperative and postoperative values of endothelial cell count and CV was done using the paired samples *T*-test. Comparisons between preoperative data and postoperative numerical data at different visits were done using repeated measures ANOVA with Bonferroni post hoc analysis. The results are considered statistically significant with a *P* value <0.05.

## 3. Results

The mean age of patients included was 56.3 ± 6.85 years (range: 35 to 72 years). Females represented 62.8% of cases, and 37.2% of cases were males. Thirty-four cases were diagnosed as POAG (79.1%), four cases were diagnosed as pigment dispersion glaucoma (9.3%), four cases were diagnosed as pseudoexfoliate glaucoma (9.3%), and one case was diagnosed as steroid-induced glaucoma (2.3%). The mean preoperative VA was 0.4 ± 0.17 (range: 0.1 to 0.8) (visual acuity was measured in decimal, converted to logMAR for statistical calculation and then converted back to decimal for presentation). Preoperative assessment of the crystalline lens revealed 5 phakic eyes and 38 pseudophakic eyes with posterior chamber IOL.

The mean preoperative IOP of the 43 patients was 31.41 ± 4.71 mmHg (range: 22 to 42 mmHg).

**Figure 3:**
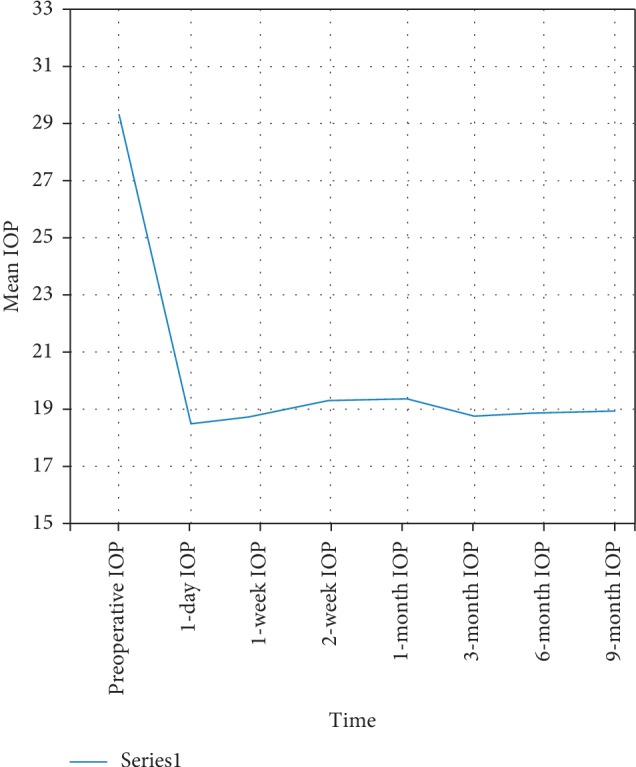
Mean IOP across postoperative visits shows a sharp decline in IOP following surgery followed by a minimal gradual rise that peaked one month after surgery followed by a further decline and then plateau phase.

The mean IOP changed across the study from 31.4 mmHg preoperatively to **18.49** ± **5.0** mmHg in the *first postoperative day*, **18.86** ± **3.4** mmHg in the *first postoperative week*, **19.44** ± **3.5** mmHg *after two weeks*, **19.55** ± **2.4** mmHg after *one month*, **18.83** ± **1.9** mmHg after *three months*, and **18.9** ± **1.8** mmHg after *six months;* by the end of study (at *nine months*), the mean postoperative IOP was **19.06** ± **1.9** mmHg (Figure 3 and Table 1). The reduction across the follow-ups was statistically significant (*P* < 0.0001) (Bonferroni corrected). The change of IOP across our study was only due to surgery except for two cases where beta blockers and carbonic anhydrase inhibitor combination was administered for the first postoperative week in one case and *α*-receptor agonist was used for the other.

**Figure 4:**
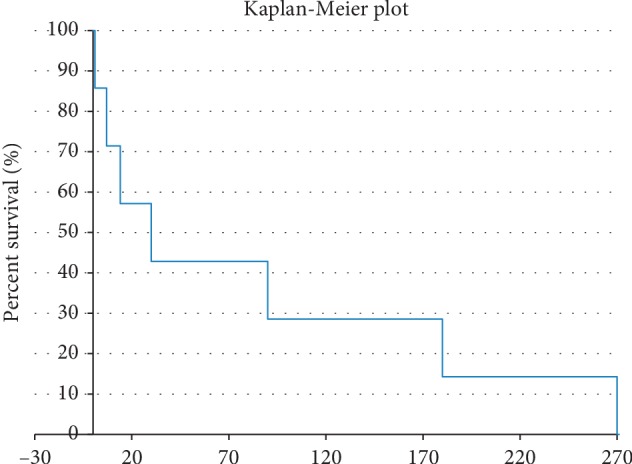
Kaplan–Meier survival curve presenting cases that fail and cases that met success criteria.

The percentage of total success was 90.7% (39 eyes). No cases met the criteria of qualified success. Four eyes (9.3%) met criteria of failure (Figure 4). The lowest postoperative IOP was 15 mmHg. The repeated measure *t*-test with two dependent means revealed a statistically significant drop in IOP between postoperative and preoperative IOP measures (*t* = 16.97, *P* < 0.05).

**Figure 5:**
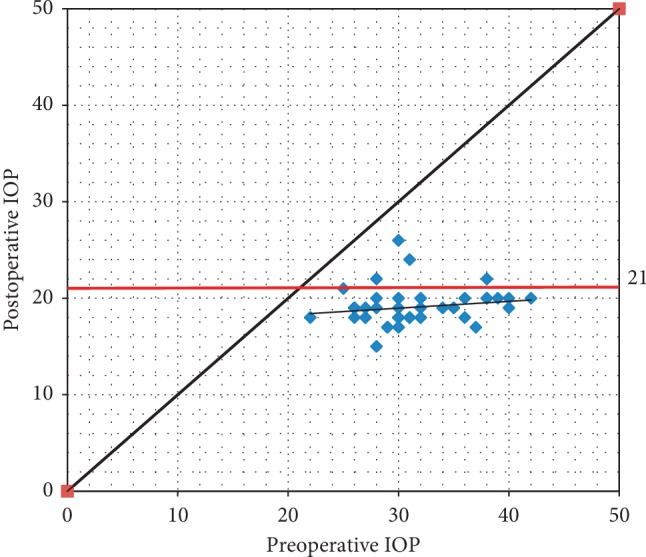
Scatter plot showing mean postoperative IOP is still lower than preoperative IOP even in failed cases.

According to the scatter gram (Figure 5), 4 cases had postoperative IOP above 21 mmHg (above the red line). Although this means that these cases are considered a failure according to our study parameters, they still lie below the diagonal line; i.e., postoperative IOP is lower than preoperative IOP.

**Figure 6:**
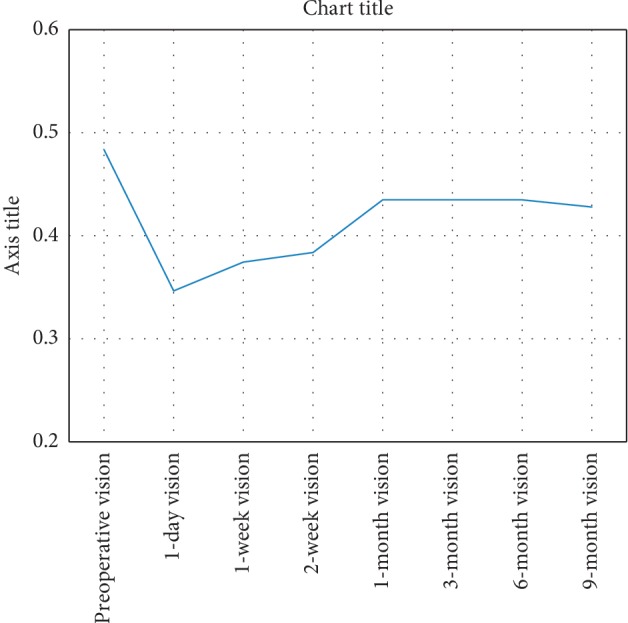
Mean VA across postoperative visits shows a sharp decline following surgery with the improvement of visual acuity gradually over the 1st postoperative month followed by a plateau phase.

The mean VA (decimal) changed across the study from baseline 0.5 ± 0.2 (range: 0.1–0.8) preoperatively to **0.3** ± **0.3** in the first postoperative day, **0.4** ± **0.1** in the first postoperative week, **0.4** ± **0.1** after two weeks, **0.4** ± **0.2** after one month, **0.4** ± **0.2** after three months, and **0.4** ± **0.2** after six months; by the end of study (at nine months), the mean postoperative VA was **0.4** ± **0.2** (range: 0.1–0.7) (Figure 6 and Table 1). There was a statistically significant drop of vision between preoperative and postoperative values (*P*=0.004).

Only 5 cases were phakic, two of them (4.65%) developed cataract, and one case (2.3%) showed increase in cataract density.

**Figure 7:**
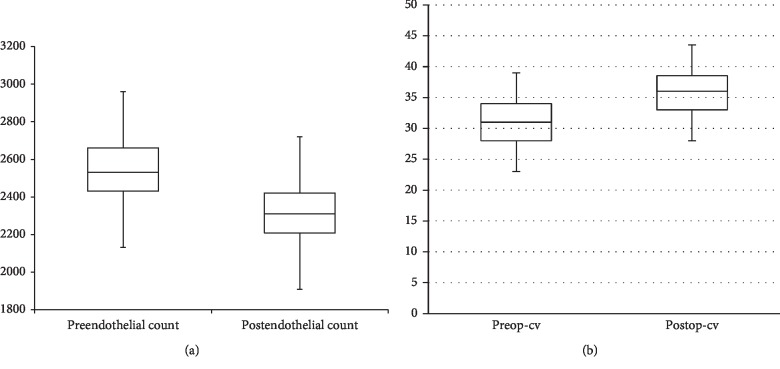
The prepostoperative change occurred in the mean endothelial count (a) and mean CV (b).

The preoperative mean central endothelial cell density (ECD) was 2551 ± 199 cells/mm^2^ (range: 2179 to 2962 cells/mm^2^). Following surgery, the mean ECD was **2320.32** ± **182** cells/mm^2^ (range: 1967 to 2710 cells/mm^2^). The repeated measure *t*-test with two dependent means reveals a significant drop in endothelial count between the postoperative and preoperative period (*t* = −17.78, *P* < 0.0001). The endothelial cell loss was **4.7%** after 9 months. The mean preoperative coefficient of variation (CV) was 31.37 ± 5.19 (range: 23 to 44). CV represents degree of variation in sizes of endothelial cells; i.e., it reflects how much cell loss is occurring. The mean postoperative CV showed a significant increase (36.02 ± 5.39) (range: 26–49) (*t* = 20.78, *P* < 0.0001), Figure 7.

No abnormality was detected in AC (no cells or flare) and pupil except for four cases. Two of them with peaked pupil (irregular). One case showed peripheral synechiae and underwent synechiolysis 17 days after the initial surgery, but synechiae reformed with an elevation of IOP that required starting alpha 2-agonist eye drops to control IOP till the end of the study. The other cases showed no synechiae but iris depigmentation at a sector shaped area adjacent to area of intervention. Only 5 cases were phakic, two showed development of cataract, and one case showed increase in cataract density.

## 4. Discussion

This study evaluates the efficacy of the short-term (9 months) follow-up of chronic open-angle glaucoma (OAG) patients treated with high-frequency deep sclerotomy (HFDS). The probe of HFDS penetrates approximately 1 mm into the sclera, through trabecular meshwork and Schlemm's canal, thus bypassing the site of resistance in trabecular meshwork [10]. Additional mechanism in lowering the IOP is the scleral thinning at the base of punctures and drainage of aqueous into the ciliary body [11]. This we can call triple mode of action. *Six* sclerotomies, in this procedure, correspond to 3.6 mm^2^ drainage surface area [12]. It only needs the abee® glaucoma tip and Oertli phaco machine. The tip is reusable and can treat thousands of cases. The technique is easy, not time-consuming, and learning curve is relatively short. Nevertheless, the studies about HFDS are still limited, and there is no study that compares its effect in lowering IOP with other MIGS procedures. However, we compared the current study results with Pajic et al. study done in 2011 and published studies for other MIGS procedures. The current study revealed significant lowering of the mean IOP following surgery from 31.4 ± 4.7 mmHg to 19.06 ± 1.9 mmHg with a success rate of **90.7%.** The study by Pajic et al. was done on 53 patients with OAG and for 72 months follow-up and revealed that mean IOP dropped from 25.6 ± 2.3 to 14.7 ± 1.8 mmHg with a success rate of 79.2%. The higher success rate in our study may be attributed to the short-term follow-up and to six sclerotomies in our study compared to four in Dr. Pajic's. Trabectome, as one of the MIGS, uses electrocautery to ablate and aspirate a portion of TM and inner layer of Schlemm's canal. Maeda et al. found that trabectome, in 80 eyes with OAG, can cause effective and safe reduction in IOP by around **28.7%** by 6 months after surgery [6]. However, Mizoguchi et al. showed that it can decrease IOP significantly by **51.8%** in 43 eyes at 2 years of follow-up [13]. The iStent Trabecular Micro-Bypass Stent works by cutting and then stenting Schlemm's canal to enhance trabecular outflow. Many randomized studies regarding the iStent were done [14, 15]. The long-term effect of second generation iStent as a standalone therapy of OAG showed **42%** reduction in IOP. Preoperative mean IOP was 25.3 ± 6.0 mmHg, postoperatively, and mean IOP was reduced to 14.6 ± 2.0 mmHg [16]. The Hydrus Microstent increases aqueous drainage into many collector channel openings as an intracanalicular dilator for Schlemm's canal and a bypass of the TM [17]. The studies, using Hydrus Microstent as a treatment for OAG patients, showed **28.5%** after one year, **38.5%** after two years in Fea et al., and *Gandofli et al.,* respectively [18, 19].

Although in the abovementioned studies, the treated eyes had lower baseline IOP than in our study, and HFDS showed a higher success rate in the short-term follow-up. This can be attributed to the special mode of action of the HFDS technique.

The current study showed a significant drop in postoperative visual acuity, which was comparable to results obtained by Pajic et al. This was not related to IOP control or glaucoma progression. Removing the effect of the three postoperative cataractous eyes, the VA improved significantly.

The safety of this technique on corneal endothelium was another important goal in our study. It was confirmed by specular microscopy to evaluate the mechanical and thermal effect of surgery on endothelium. Comparing different MIGS techniques regarding ECL showed that Hydrus Microstent has the highest mean % of loss, followed by iStent and HFDS (in our study), and trabectome showed the least ECL, **14%** in 24 months, **13.1%** in 24 months, **4.7%** in 9 months, and **no change** in 12 months, respectively. HFDS in current study comes second after trabectome in preserving ECD. Nevertheless, no postoperative corneal edema or any clinical sequelae were observed in our study. The significant ECL in this study may be attributed to the compromised endothelium in pseudoexfoliate glaucoma patients (4 cases).

High-frequency deep sclerotomy is a safe and promising minimally invasive procedure which successfully achieves a lower IOP for chronic open-angle glaucoma patients. Long-term follow-ups and greater number of cases is needed before we can decide to add this procedure to the current glaucoma management regimen and determine its position. *The main disadvantage of HFDS is that it leads to midteens in IOP which is not adequate in advanced glaucoma patients*. Theoretically, it does not interfere with further laser or penetrating glaucoma surgery as the conjunctiva is spared. Further studies about the effect of repeating this procedure twice or thrice in cases we needed more IOP control is recommended. Also, another study should be done is the effect of this procedure in vitrectomized patients, where the resultant conjunctival fibrosis after vitrectomy limits the success of other glaucoma surgeries. Three of phakic eyes showed limited IOP reduction as the only two cases that showed irregular pupils were phakic with one eye showing synechiae (as was mentioned before). But the number of phakic eyes was not reliable in this study to reach a conclusion about the efficacy of HFDS in phakic patients compared to pseudophakic ones.

HFDS is one of the promising MIGS procedures that lack proper research.

## Figures and Tables

**Table 1:** Mean difference and statistical significance between preoperative values (IOP and VA) and postoperative follow-ups.

Preoperative value (mean and range)	Postoperative follow-up	Mean value	Standard deviation	Range	Mean difference	*P* value^♠^
Mean IOP **31.4**, range 22–42 mmHg	1 day	**18.5**	5	8–30	12.9	<0.0001
	1 week	**18.9**	3.4	14–30	12.6	<0.0001
	2 weeks	**19.4**	3.5	12–30	11.9	<0.0001
	1 month	**19.6**	2.4	16–26	11.9	<0.0001
	3 months	**18.8**	1.9	15–25	12.6	<0.0001
	6 months	**18.9**	1.8	15–25	12.5	<0.0001
	9 months	**19.1**	1.9	15–26	12.3	<0.0001

Mean VA **0.48,** range 0.1–0.8 decimal	1 day	**0.3**	0.3	0.1–2	0.1	0.0814
	1 week	**0.4**	0.1	0.1–0.6	0.1	<0.0001
	2 weeks	**0.4**	0.1	0.1–0.6	0.1	<0.0001
	1 month	**0.4**	0.2	0.1–0.7	0.04	0.0041
	3 months	**0.4**	0.2	0.1–0.7	0.04	0.0041
	6 months	**0.4**	0.2	0.1–0.7	0.05	0.0041
	9 months	**0.4**	0.2	0.1–0.7	0.06	0.0044

^♠^Bonferroni corrected.

## Data Availability

The data of this manuscript were restricted by the ethics committee of the Research Institute of Ophthalmology (R.I.O), Cairo, in order to protect patient privacy. Data are available from Dr. Shaymaa Hassan Salah, shaimaa.sayed@kasralainy.edu.eg, for researchers who meet the criteria for accessing confidential data.
